# Is pathological aging a successful resistance against amyloid-beta or preclinical Alzheimer’s disease?

**DOI:** 10.1186/alzrt254

**Published:** 2014-05-06

**Authors:** Melissa E Murray, Dennis W Dickson

**Affiliations:** 1 Department of Neuroscience, Mayo Clinic, 4500 San Pablo Road, Jacksonville, FL 32224, USA

## Abstract

Individuals with pathological aging, a form of cerebral amyloidosis in older people, have widespread extracellular amyloid-beta (Aβ) senile plaque deposits in the setting of limited neurofibrillary tau pathology. Unlike the characteristic finding of antemortem cognitive impairment in Alzheimer’s disease patients, individuals with pathological aging usually lack cognitive impairment despite similar Aβ senile plaque burdens. It has been hypothesized that protective or resistance factors may underlie pathological aging, thus minimizing or preventing deleterious effects on cognition. Despite increasing interest and recognition, a review of the literature remains challenging given the range of terms used to describe pathological aging. This debate briefly reviews neuropathologic and biochemical evidence that pathological aging individuals have resistance factors to Aβ plaque pathology. Additionally, we will discuss evidence of pathological aging as an intermediate between normal individuals and Alzheimer’s disease patients, and discuss protective or resistance factors against vascular disease and neurofibrillary pathology. Lastly, we will emphasize the need for longitudinal biomarker evidence using amyloid positron emission tomography, which will provide a better understanding of the kinetics of Aβ deposition in pathological aging.

## Introduction

Histopathologic changes characteristic of Alzheimer’s disease (AD) have been described in nondemented older individuals [1-7], with particular focus on a form of senile cerebral amyloidosis – termed pathological aging. The neuropathologic characteristics of pathological aging are extracellular amyloid-beta (Aβ) in senile plaques [8] and intracellular tau in neurofibrillary tangles and neuropil threads. In AD these lesions are accompanied by dendritic and synaptic abnormalities, as well as neuronal loss. In pathological aging, however, significant Aβ deposits (sufficient for a neuropathologic diagnosis of AD using Khachaturian criteria [9]) are detected in the setting of minimal cortical and limbic neurofibrillary pathology [2].

Pathological aging is usually a finding in older individuals who have no significant antemortem cognitive impairment, and some individuals may even be high functioning. Whether it is preclinical AD is controversial. Without longitudinal biomarker evidence to ascertain the timing of amyloid deposition, it is uncertain how long amyloid deposits have been present in the brains of individuals with pathological aging. Recently, it was suggested that such individuals may have protective or resistance factors against the neuronal and synaptic pathology, which are structural correlates with cognitive impairment. While increasingly recognized, there is a range of terms for pathological aging that makes review of the literature challenging, including presymptomatic or incipient AD [4], preclinical AD [6], pathological AD [7], nondemented high pathology controls [3], asymptomatic persons with AD neuropathology [10], intermediate probability mismatches [11], and pathologically preclinical AD [5]. This debate will briefly review the evidence for and against the notion that pathological aging is a state of resilience from the putative toxic effects of Aβ.

### Evidence supporting a resistance to amyloid-beta in pathological aging

Some of the strongest data that support the hypothesis of a resistance factor in pathological aging come from neuropathologic and biochemical studies. Early investigations of pathological aging revealed that although senile plaque density was sufficient for AD according to Khachaturian criteria [9], morphologic and immunohistochemical differences existed between Aβ deposits in pathological aging and AD. Diffuse plaques, principally composed of Aβ42 [12], were more abundant in pathological aging, and paired helical filament tau immunoreactivity or glial reaction was rarely observed in proximity to senile plaques [2,4]. Moreover, Aβ deposits in pathological aging had less codeposition of apolipoprotein E and the individuals had less advanced glycation end product immunoreactivity [13,14]. Biochemical analyses of Aβ peptides in pathological aging and AD revealed overlapping profiles [15]. Subtle quantitative differences were noted, but evidence from mass spectrometry identified more amino-terminal truncations in Aβ of AD compared with pathological aging. Aβ42 levels, however, have been found elevated in pathological aging compared with AD [3,15], which probably represents greater abundance of diffuse plaques in pathological aging [12].

Oligomeric assemblies of soluble Aβ pools were not different, but insoluble Aβ – particularly Aβ in formic acid and guanidine hydrochloride extracts – better distinguished pathological aging from AD. The data supported previous findings from a study investigating neuropathologic and biochemical differences in the oldest old, which evaluated nondemented pathological aging (referred to as high pathology controls) [3]. When compared with an age-matched, demented AD cohort, soluble oligomeric assemblies were not found to differ. A recent study evaluating differences in normal individuals, pathological aging (referred to as intermediate probability mismatches), and AD confirmed these findings [11], but added new information suggesting that Aβ oligomeric monomers and dimers in synaptoneurosomes may be lower in nondemented pathological aging compared with demented AD. Using array tomography to visualize and quantify synaptic densities near amyloid deposits in AD, Koffie and colleagues provide evidence for deleterious effects of oligomeric Aβ [16]. They further show that synapse loss is exacerbated in apolipoprotein E ϵ4 carriers, who also had higher levels of oligomeric Aβ. Given the extensive overlap between AD and pathological aging with respect to soluble oligomeric assemblies [15], but reportedly greater synaptophysin and neprilysin levels in pathological aging [17], an unknown resistance factor may mitigate the effects of oligomeric Aβ species on synaptic pathology.

### Evidence opposed to a resistance to amyloid-beta in pathological aging

Early biochemical studies evaluating insoluble and soluble Aβ40 and Aβ42 provide evidence for an alternative hypothesis – that pathological aging is merely a transitional state between normal aging and AD [18]. Using an enzyme-linked immunosorbent assay to measure pools of Aβ, the largest soluble Aβ1–40 and Aβ1–42 pools were found in the brains of nondemented normal individuals (50% and 23%, respectively), and the smallest fraction was found in demented AD brains (2.7% and 0.7%). Soluble Aβ1–40 and Aβ1–42 were intermediate in nondemented pathological aging (8% and 0.8%, respectively), whereas the insoluble Aβ1–40 pool in AD was found to be 20-fold higher than in pathological aging brains.

Rather than a resistance factor to Aβ, a third hypothesis postulates that factors other than Aβ mediate cognitive deficits in AD, most notably effects of cerebrovascular disease and neurofibrillary tau pathology. When comparing AD with pathological aging, white matter rarefaction and cerebral amyloid angiopathy were found to be more severe in AD [3]. These findings suggest that white matter pathology associated with reduced perfusion due to cerebral microvasculature dysfunction may contribute to differences in cognitive dysfunction between the two groups. Although inherent to the classification of pathological aging, less neurofibrillary pathology was shown to correlate with better cognitive performance in pathological aging (referred to as asymptomatic persons with AD neuropathology) [10]. Lower vascular risk and older age were also found to associate with pathological aging compared with demented AD patients. Further supporting this third hypothesis, a recent study investigating a nondemented cohort found that vascular risk factors (for example, diabetes, hypertension) influenced the relationship among memory, cortical thickness, and Aβ as measured using [^11^C] Pittsburgh compound B positron emission tomography [19].

### Explanations for conflicting evidence

One of the largest hurdles in addressing this controversy is the different constructs used by various investigators to describe pathological aging. Each of the three hypotheses presented was based on data that did not conflict as much as provide supportive evidence that multiple factors contribute to cognitive deficits associated with AD pathology. In understanding the significance of Aβ in cognitively normal individuals, it is important to consider that death due to intercurrent illnesses (for example, cancer, heart disease, respiratory infection) may truncate the disease process at a point where it is impossible to know whether dementia would have been the ultimate outcome had accumulation of Aβ led to neuronal and synaptic damage. Without knowledge of the kinetics of amyloid deposition in pathological aging, it will be difficult to determine whether there are actually factors that confer protection (for example, genetic variants, cognitive reserve) to toxic effects of Aβ accumulation.

## Conclusion

The main issue one should consider is where patients with pathological aging lie in the course of amyloid deposition, whether this is an acute process or one that has been playing out over a number of years. Efforts have been made to establish the age of Aβ proteins through investigations into post-translational modifications such as N-terminal degradation, racemization, isomerization, oxidation, pyroglutamyl formation, and covalently linked dimers [20,21]. Neuropathologic studies can classify Aβ, tau and other co-existing pathologies, but it is not yet possible to determine with certainty the age at which Aβ deposition began in an individual patient. Through the use of longitudinal amyloid positron emission tomography imaging, we will be able to better address whether pathological aging patients are resistant to the deleterious effects of long-standing Aβ deposition.

## Abbreviations

AD: Alzheimer’s disease; Aβ: Amyloid-beta

## Competing interests

The authors declare that they have no competing interests.
